# A Brief Review of the EEG Literature on Mindfulness and Fear Extinction and its Potential Implications for Posttraumatic Stress Symptoms (PTSS)

**DOI:** 10.3390/brainsci9100258

**Published:** 2019-09-27

**Authors:** Auretta S. Kummar, Helen Correia, Hakuei Fujiyama

**Affiliations:** Discipline of Psychology, Exercise Science, Chiropractic and Counselling; College of Science, Health, Engineering and Education, Murdoch University, Murdoch 6150, Australia; helen.correia@murdoch.edu.au (H.C.); Hakuei.Fujiyama@murdoch.edu.au (H.F.)

**Keywords:** mindfulness, fear extinction, trauma, PTSD, electroencephalogram (EEG), neurofeedback

## Abstract

Neuroimaging studies in the area of mindfulness research have provided preliminary support for the idea of fear extinction as a plausible underlying mechanism through which mindfulness exerts its positive benefits. Whilst brain regions identified in the fear extinction network are typically found at a subcortical level, studies have also demonstrated the feasibility of cortical measures of the brain, such as electroencephalogram (EEG), in implying subcortical activations of the fear extinction network. Such EEG studies have also found evidence of a relationship between brain reactivity to unpleasant stimuli (i.e., fear extinction) and severity of posttraumatic stress symptoms (PTSS). Therefore, the present paper seeks to briefly review the parallel findings between the neurophysiological literature of mindfulness and fear extinction (particularly that yielded by EEG measures), and discusses the implications of this for fear-based psychopathologies, such as trauma, and finally presents suggestions for future studies. This paper also discusses the clinical value in integrating EEG in psychological treatment for trauma, as it holds the unique potential to detect neuromarkers, which may enable earlier diagnoses, and can also provide neurofeedback over the course of treatment.

## 1. Introduction

Interest and research into the area of mindfulness have had a steep growth trajectory in recent decades. Owing to this is the development of various mindfulness-based interventions as well as the growing body of evidence on its efficacy for a broad range of populations—be it to improve general well-being amongst healthy individuals [1,2], or in the treatment of clinical disorders such as depression and anxiety [3,4], chronic pain [5,6], insomnia [7,8], and substance abuse [9,10].

This surge in popularity has been paralleled in the media, with some authors criticizing the “hype” especially with regard to the overestimation of its clinical effectiveness [11]. Arguably, this could also be perceived as true for any research area and/or intervention that is still in its inception; and as such, it demonstrates the invaluable theoretical and clinical importance in balancing the existing knowledge and claims of mindfulness with scientific and clinical evidence. Initiatives toward this end include neuropsychological studies that seek to explore the underlying mechanisms through which mindfulness may be yielding its suggested positive effects [12,13,14,15,16,17,18].

Across the literature, there may be slight variations as to how specific mechanisms of mindfulness are referred to but, in general, the proposed theoretical framework of mindfulness is suggested to include (i) attention regulation, (ii) body regulation, (iii) change in perspective of self, and (iv) emotion regulation [16]. Within this framework by Holzel et al. [16], ‘emotion regulation’ was conceptualized to consist of (a) reappraisal, and (b) exposure, extinction, and reconsolidation, whereby the former refers to the non-judgmental response to emotions, and the latter refers to the process of being exposed to and consciously affected by adverse experiences, but without responding reactively whether through physical symptoms, thoughts, or feelings.

Although these various mechanisms have been suggested, it could be perceived that the strength of the evidence base differs between one suggested mechanism to another. For instance, there are relatively more studies (although also, this is still a growing research area) that have investigated attention regulation as a mechanism of mindfulness, in comparison to that in exploring the role of “exposure, extinction, and reconsolidation” (i.e., fear extinction) in mindfulness [19,20,21]. As such, there is arguably a need to further explore fear extinction as one potential underlying mechanism of mindfulness, as this could hold important implications for the use of mindfulness-based interventions as an evidence-based practice for fear-based disorders such as anxiety, phobias, and some responses to trauma [22].

The idea of fear extinction as a process that underlies mindfulness has been suggested in several papers [12,20,21], and was the focus of a recent review [22]. Specifically, in the review, parallel findings in the neuroimaging literature of fear extinction and mindfulness were discussed; emerging evidence was argued to hold important implications for trauma-based psychopathologies. It also discussed the importance of corroborating client-reported and/or clinician-rated effectiveness of mindfulness-based interventions with neuropsychological measures to augment the current literature, as this could fully characterize how mindfulness may facilitate fear extinction. Therefore, the present paper aims to provide a brief conceptual review that expands the neuropsychological context of this argument into the neurophysiological literature of mindfulness and fear extinction—particularly, that utilizing electroencephalogram (EEG) measures. The basis for specifically extending this argument into the EEG literature stems from considerations around the feasibility, clinical significance, and cost-effectiveness of EEG measures in contrast to neuroimaging techniques.

Whilst neuroimaging techniques hold considerable contribution in advancing the theoretical and empirical knowledge of mindfulness, it presents formidable challenges from a clinical perspective, as neuroimaging techniques are not always available in clinical settings. Where there are, they may be a costly assessment for patients. Moreover, neuroimaging tools, such as the magnetic resonance imaging (MRI) scanner, have the potential to induce claustrophobic anxiety amongst patients, which in turn, interferes with treatment progress [23]. This is in contrast to the use of the EEG, which is a non-invasive, neurophysiological method of passively monitoring and recording electrical activity in the brain (i.e., brain waves).

Moreover, as the EEG is arguably more feasible and cost-effective, it holds greater clinical significance to be incorporated as an adjunct neurofeedback treatment in mental health settings [24]; this neurofeedback has been demonstrated with specific regard to enhancing mindfulness-related capacities [25]. Put together, there is likely to be theoretical, empirical, and also clinical benefits, in understanding the neurophysiological (i.e., EEG) workings and relationships between fear extinction and mindfulness. Therefore, this paper puts forward this seminal step by providing a brief discussion on the following: (i) mindfulness, (ii) fear extinction, including neural correlates via neuroimaging and neurophysiological techniques, (iii) the link between mindfulness and fear extinction as illustrated through EEG findings on mindfulness, (iv) implications for trauma, and (v) future studies in this area.

### 1.1. Mindfulness

Historically, mindfulness has its roots in the 2500-year-old spiritual practice of Buddhism. However, it was not until the early 1980s that mindfulness was translated into a Western, non-religious context as a practice or a technique. Since then, mindfulness meditation has commonly been described as the awareness and attention that is directed purposefully in a non-judgmental manner from one moment to the next [26]. There are a number of ways through which mindfulness can be cultivated, such as, through the mindful exercise of Qigong, Tai Chi, and/or yoga [27,28]. However, when reported in a research or clinical context, mindfulness meditation has been given predominance as an approach to developing mindfulness [12,29].

In a review by Lutz, Slagter, Dunne, and Davidson [30], neuropsychological evidence was discussed in support of the theoretical framework they suggested—that is, mindfulness meditation that encompassed two forms of meditation, specifically, focused attention meditation (FA) and open monitoring meditation (OM). FA involves the deliberate focus of attention on an object (e.g., a sensation caused by breathing) with the recognition, and thus refocusing, of attention back toward the object as and when the mind wanders. OM, on the other hand, is practiced after the initial use of FA and entails the non-reactive monitoring of experiential phenomena (i.e., the physiological sensations, thoughts, and/or emotions), moment to moment, without an explicit focus on any specific object. In this way, mindfulness meditation takes on a non-reactive stance or approach toward interoceptive experiences, including aversive emotions and memories. Associated forms of meditations—specifically, loving-kindness/compassion-focused meditations—have also been suggested to incorporate both FA and OM meditations [21,31].

Stemming from the concept and practice of mindfulness meditation, various mindfulness-based interventions have been developed, including the well-established mindfulness-based stress reduction program (MBSR) [32,33] and mindfulness-based cognitive therapy (MBCT) [34,35], which are frameworks that have in turn influenced the development of other mindfulness-based interventions (e.g., mindfulness-based relapse prevention for substance abuse and mindfulness-based eating awareness therapy for binge eating) [36,37]. Another such mindfulness-based intervention that has been introduced specifically to the area of trauma is mindfulness-based exposure therapy; however, this will be explored toward the end of this paper.

### 1.2. Fear Extinction

A brief background to the construct of fear extinction has been presented in a former paper [22], and also more broadly elsewhere [38]. However, it should be further reiterated that fear extinction does not imply the unlearning of the association formed between the neutral, unconditioned stimuli and the feared, conditioned stimuli. Instead, fear extinction has been argued to imply the learning of a new memory that competes without erasing the original fear memory [38,39]; alternatively, it has been suggested to imply the reconsolidation of the original fear memory with new contextual associations [40,41]. The differences between these two conceptualizations can be best understood with respect to the findings by Gershman et al. [42]. Particularly in their study, rats who experienced an ‘abrupt’ extinction (i.e., removing the feared stimuli all at once) were suggested to have formed a new, competing memory that weakened over time and gave rise for the original, fear memory to resurface again. This is in contrast to rats who experienced gradual extinction (i.e., by gradually removing the feared stimuli), whereby the original fear memory was suggested to have been modified as opposed to forming a new competing memory. As a result, the rats who experienced a gradual removal of the feared stimuli had significantly lower rates of experiencing a return in symptoms following a lapse in duration. The understanding of this extinction paradigm could perhaps suggest a framework for exposure work with humans—specifically, by supporting humans gradually develop coping strategies that they can practice whilst being exposed to the feared stimuli, which hypothetically may then modify the original fear memory over a series of clinical sessions. In the context of mindfulness, these strategies would include cultivating mindful attention and awareness of thoughts, emotions, and bodily sensations when exposed to the stimuli or when they are brought to mind, whilst mindfully responding with non-reactivity, curiosity, and non-judgment; as opposed to triggering a reactivation of the threat system, or waiting out the threat response when exposed to the feared stimuli. This is further discussed later in this paper, in relation to the link between mindfulness and fear extinction.

#### 1.2.1. Neural Correlates of Fear Extinction

The neuropsychological mechanisms of fear extinction have been reviewed extensively in several reviews elsewhere [38,43,44], and were also briefly reviewed in a recent paper [22]. Of note, implicated brain regions include the amygdala (i.e., the brain region associated with emotional processing, including that of fear expression), the hippocampus (i.e., the brain region involved in memory consolidation and reconsolidation, and thus, in signaling the safety context of extinction), and the ventromedial prefrontal cortex (vmPFC; i.e., the brain region instrumental in decision making and emotion regulation, including the processing of risk and fear). Collectively, these brain regions have been implied in fear extinction through the harmonious down-regulation of the amygdala by the vmPFC and the hippocampus [45,46].

#### 1.2.2. Neurophysiological Literature on Fear Extinction using EEG

Whilst the fear extinction network has typically been implied in subcortical brain regions using neuroimaging studies, neurophysiological studies utilizing EEG have also been employed. The ability of the brain to link an aversive stimulus to a neutral stimulus (which becomes a conditioned, fear stimulus) was theorized on early principles of association [47]. According to this Hebbian principle, the linking process is initiated when a neuron continuously contributes to the firing of another, and that the synchronous activation of two neurons (or neuron systems), which may lie closely next to each other (i.e., a millimeter in range) or in distinct cortical lobes, strengthens the connection between them. Advances in neuroscience since then have been able to largely validate this theory (for a review, see [48]).

EEG analyses of brain activity are mainly grouped into two categories: the time domain or frequency domain of EEG. The former typically utilize event-related potential (ERP), which is the measure of brain response that is time-locked to the onset of an event (e.g., a sensory stimulus). ERPs reflect the EEG activity that is evoked by the presented stimuli/event. The frequency domain analyses of EEG include the analyses of spectral power (i.e., the magnitude of a measured signal against its frequency), event-related synchronization and desynchronization (ERS/ERD; i.e., a relative increase and decrease in power, respectively), as well as coherence/synchronization across brain regions (i.e., sources of brain activity that are approximately phase-locked with each other). Additionally, by adopting a source localization technique via, for example, low-resolution electromagnetic tomography (LORETA) [49], the sources of brain activity associated with a certain event may be implicated.

##### Event-Related Potentials

Studies using ERPs have demonstrated increased P300, which is a positive deflection in voltage with a latency of approximately 250–500 ms, in response to emotional stimuli, including threatening visual or auditory stimuli [50,51,52,53]. The P300 has been described to play a role in the processing of the stimulus context as well as levels of attention and arousal [54,55]. More specifically, the P300 family is made up of interacting subcomponents, P3a and P3b. P3a originates from frontal distribution to reflect stimulus-driven attention or working memory during task processing, whereas P3b originates from temporal–parietal distribution to reflect attention associated with memory-updating processes, and is relevant for future memory processing [56]. Accordingly, it has been suggested that whilst P3a is related to task-irrelevant distractors, only P3b is related to the valence or arousal of targets [52]. Therefore, in combination, robust evidence appears to suggest a hippocampal origin for the P300 potential, although the relative contribution of the hippocampus to the P300 potential is less clear [54].

The P300 is also one of the ERP components that makes up a cluster referred to as the late positive potentials (LPP). Whilst there might be slight variations across studies as to what constitutes the LPP, it is typically computed as the average amplitude within the time window of 300–600 ms after a stimulus, across central (C3, C4, and Cz), parietal (P3, P4, and Pz), and occipital (O1, O2, and Oz) sites [52]. Similar to the role of P300, the LPP has been suggested to reflect the deeper and motivated processing of emotional information [57,58,59,60]. As such, it is almost expected that the LPP has also routinely been implied in the processing of emotionally salient stimuli, including those that imply threat [50,51,57,61]. In combination, the P300 and LPP potentials allude to an overarching motivated attentional process to arousing stimuli, which may in part (and not exclusively) be threatening.

##### Source Localization

As stated earlier, fear extinction is typically implied with activations of subcortical brain regions. As such, source localization analyses with EEG measures may be helpful in implying the subcortical regions that are involved—although it should also be noted that such source-based EEG analyses ought to be interpreted with caution [62,63].

Alterations in vmPFC-localized (infralimbic in rodents) gamma activity were indicated in the extinction of conditioned fear, whilst anterior cingulate cortex (ACC)-localized (prelimbic in rodents) theta activity has been associated with the expression of conditioned fear [64,65,66] (the studies by Fenton et al. [64,65] were conducted with rats, and therefore resulting findings in prelimbic and infralimbic cortex were suggested as the rodent homologs of the ACC and vmPFC in humans, respectively). Altered gamma activity, which was found in Mueller et al., [66] was also found in the left hippocampus—a region that, as indicated earlier, is implied in the recall of fear extinction. The findings from Mueller et al. [66] have been notable as the study was conducted with humans, and therefore is an impetus to elucidating the valuable use of EEG in fear extinction research in humans.

In the amygdala, theta activity has been implied in response to emotional arousal [67], including stimuli with a negative valence (e.g., threatening stimuli). Theta activity has also been suggested to couple with gamma activity in the amygdala during fear expression and extinction. Specifically, in periods of fear, theta–gamma coupling in the amygdala was enhanced, while gamma power was suppressed [68]. On the contrary, periods of relative safety were related to an enhanced amygdala-localized gamma power, which showed a medial PFC–amygdala directionality and was also found to be a consequence of theta activity in the medial PFC. Together, these findings suggest that amygdala-localized gamma activity couples with amygdala-localized theta activity during fear expression, and medial PFC-localized theta activity during fear suppression.

Moreover, by combining multiple site local field potential, studies conducted with mice found evidence of coupled theta activity in the amygdala–hippocampus–PFC cortical circuits during fear extinction [69,70]. Findings by Lesting et al. [70] were further able to demonstrate a direction in this theta interaction, with PFC-localized activity in the lead of hippocampal-localized and amygdala-localized theta activity. The finding of an interaction between these regions is supported by functional neuroimaging studies, which similarly show the vmPFC, the hippocampus, and the amygdala to be implied in the fear extinction network, as discussed above [45,46].

### 1.3. Link between Fear Extinction and Mindfulness

Experiential avoidance—that is, the intolerance and/or maladaptive efforts to avoid distressing thoughts, emotions, and/or physiological sensations—plays a central role in the maintenance of learnt fear [71]. In contrast to this, the practice of mindfulness encourages a non-judgmental and non-reactive monitoring of those distressing experiences within one’s conscious awareness.

Accordingly, it has been suggested that the conscious awareness of one’s aversive thoughts, emotions, and/or bodily sensations, concurrent with the non-reactive response toward them, may desensitize the aversive strength of those experiences, leading to the extinction of a fear response toward them [12,17,34]. In other words, mindfulness encourages an extinction of the feared response by altering how we relate to and experience the feared stimuli—that is, by embodying mindful attention and awareness of thoughts, emotions, and bodily sensations when exposed to the stimuli, and then, by mindfully choosing to respond to the stimuli with curiosity and non-judgment, as opposed to reacting to the stimuli with an automatic, fight/flight response. From this perspective, mindfulness has been stated to demonstrate similarities with the concept of ‘exposure and avoidance prevention’ seen in exposure therapy, and has therefore been proposed as a form of psychological exposure [20].

Mindfulness also differs from mere habituation in the process of fear extinction, such that it cultivates increased self-awareness, non-judgment, and curiosity aspects, which may arguably enhance the modification of the original feared memory, as discussed in the context of the findings by Gershman et al. [42]. To elaborate, the pairing between the neutral (e.g., loud bang) and objectively safe conditioned stimulus (e.g., setting) that typically results in a conditioned fear response would now be modified with an ability to be aware of and describe interoceptive responses (i.e., thoughts, emotions, and bodily responses), in a non-judgmental, non-reactive, and curious manner, resulting in a positive shift in the conditioned response.

#### 1.3.1. Neurophysiological Literature on Mindfulness Using EEG

Recent efforts have been made to review the neuroimaging findings of mindfulness with respect to exploring the link between mindfulness and fear extinction [22]. Similarly, this paper strives to further explore this link, but with studies utilizing EEG methods instead. Table 1 illustrates the various EEG studies on mindfulness conducted with non-clinical samples that are reviewed in this section. It is noted that the findings summarized here are only those that are deemed relevant to the purpose of the current paper in understanding the link between fear extinction and mindfulness.

##### Event-Related Potentials

Implicated ERPs that have recurrently been found in studies that have investigated the mechanisms and/or effects of mindfulness are P300 and LPP [92]. With specific regard to P300, studies have found mindfulness to be associated with an increase in P300 in response to targeted stimuli [73,80,84,90,91] and a decrease in P300 in response to distractor stimuli [73,88] or in association to higher self-reports of ‘decentering’ [81]. These results have in turn led to the idea of efficient distributed attention in mindfulness-based meditations, whereby meditators are better able to allocate attention between relevant and irrelevant stimuli as demanded by the task [73,90].

van Leeuwen et al. [90] specifically demonstrated this by showing that among Zen meditators (comprising of both FA and OM), meditators had increased attention to small, detailed targets in comparison to controls with no meditation experience (the results mentioned here are those relevant to the P300 only. Complete results indicate that meditators processed small stimuli (embedded within a larger stimuli) at P1, N2, and P3, in comparison to controls, who only processed small stimuli at P1. Similarly, meditators processed large stimuli (that were made up of the smaller stimuli) at N1, N2, and P3, in comparison to controls, who only processed large stimuli at P3. Together, this indicates a greater ability among meditators to engage and disengage attention between spatial locations.). However, following a four-day OM-only based meditation, meditators with extensive FA meditation experience had reduced capacity to attend to the small, detailed targets, from pre-retreat to post-retreat. van Leeuwen et al. thereby concluded that whilst FA-based meditations cultivate the focusing of attention to expected stimuli, OM-based meditations train a more distributed attention with the ability to allocate and reallocate attention in response to the demands of a task.

It is acknowledged that these findings pertain to the effects of mindfulness on attention regulation, whereby these findings specifically suggest the improved allocation of attention as indexed by increased P300 to relevant stimuli and a decrease in P300 to irrelevant stimuli. However, it is arguable that these findings may hold clinical relevance in the context of fear extinction—particularly, with how attention is allocated to arising stimuli in a fear context. To elaborate further, these findings, which suggest an improved allocation of attention following mindfulness, might imply that mindfulness-based meditations and practices may be helpful in cultivating and strengthening the skill of detaching or disengaging from arising stimuli that may otherwise trigger a threat-based reaction that could narrow one’s focus of attention on that particular target or feared stimuli. Stemming from this assumption, it would therefore be interesting to investigate how mindfulness training might alter these attentional resources in the fear extinction context, where attention toward an initially feared stimulus is expected to decrease, and would therefore be indicated by a decrease in P300.

Given the overlap between the P300 and LPP in reflecting deeper and motivated processing of emotional information as described earlier, the LPP have also been indicated in EEG studies on mindfulness [92]. In particular, an inverse correlation has been found between dispositional mindfulness and LPP in view of unpleasant and highly arousing images [77]. Similarly, findings by Sobolewski, Holt, Kublik, and Wróbel [89] have found meditators to experience lower LPP in response to negative valence stimuli, but were no different from controls in response to positive valence stimuli, suggesting that meditators were better able to regulate negatively arousing emotions. On the other hand, Egan, Hill, and Foti [82] found increased LPP regardless of affective valence and arousal. Egan et al. [82] attributed this finding to the nature of their study, such that the brief mindfulness instruction in their study requested participants to focus their attention to external stimuli (pictures on the screen), which would have in turn led to increased LPP to reflect emotional processing of the stimuli to which focus was directed. As such, the ERP findings thus far allude to the role of mindfulness meditation in the motivated allocation of attention resources, which could have important implications for how attention is allocated toward feared stimuli in the context of fear extinction.

##### Spectral Power and Coherence

Further neurophysiological evidence on mindfulness typically suggests an increased oscillation in alpha and theta frequencies [92,93,94]. Together, increased alpha and theta oscillations, with the latter mostly occurring in the frontal midline region (which includes the PFC and ACC), have been suggested to imply enhanced attentional processing toward internalized stimuli [92,94].

However, of interest to the current paper are alterations in gamma and theta activity, which as elaborated under “Neurophysiological literature on fear extinction using EEG”, have been found to be associated with fear extinction and expression respectively. With specific attention to gamma activity, the majority of mindfulness-based studies have found an increase in gamma activity [72,76,78,79,83,85,86,87]. Increases in gamma power have further been revealed to be positively associated with years of meditation [76,78,86].

Yet, there has also been evidence of decreased gamma activity following mindfulness meditation [74,75]. In their study, Berkovich-Ohana et al. [75] found that the deactivation of the default mode network (DMN—the network associated with mind wandering) is indicated by a reduced overall inter-hemispheric gamma mean phase coherence when transitioning from resting state to a time production task, and therefore concluded their results to suggest a reduction in mind wandering in higher trait mindfulness (interhemispheric phase coherence refers to the alignment of oscillatory phases between homologous cortical regions (e.g., the left and right dorsolateral prefrontal cortex). In the context of the results by Berkovich-Ohana et al. [75], the homologous cortical regions are the entire hemispheres). These results are also better understood when examined with respect to spectral power. Particularly, where the deactivation of the DMN was identified as a decrease in gamma power over frontal and midline regions, meditators showed lower trait frontal gamma power, indicating lower mind wandering. However, meditators were also found with greater trait and state posterior gamma power, which was attributed to greater attentional skills and awareness of arising interoceptive and external stimuli [74]. These findings in the posterior regions have similarly been found in other studies as well [76,78,79].

Interestingly, Lutz et al. [86] found a ratio between gamma and theta activity, whereby, in contrast to controls, long-term practitioners were found to display a higher ratio of gamma to low frequency bands (i.e., theta and alpha bands) at an initial resting state, which was then enhanced during meditative practice and maintained at a post-meditation resting state. Long-term practitioners were also found to have a larger size of gamma synchrony patterns over lateral frontoparietal regions in comparison to controls; and that increases in synchrony size when shifting from resting to meditative states were greater for meditators than control. These findings could possibly reflect changes in attentional and affective processes as a result of mindfulness practice. Additionally, this ratio could also suggest an interactive role between gamma and theta activity, which has previously been discussed, are also implied in the extinction and expression of fear, respectively.

Also worth noting are findings by Milz et al. [87], which elucidated the difference between conventional, head-surface coherence and intracortical lagged coherence that utilizes EEG tomography. Specifically, Milz et al. concluded that functional connectivity using conventional, head-surface coherence shows increases in coherence (which included increased gamma coherence), whereas functional connectivity using intracortical lagged coherence resulted in decreases in coherence (which included lowered theta coherence). Milz et al. argued that in line with the findings by Lehmann et al. [85], there may be no association between conventional, head-surface coherence and intracortical lagged coherence, and that a comparison of results yielded between head-surface conventional coherence and intracortical lagged coherence may not be possible. Of note, Miltz et al. interpreted the decreases in intracortical lagged coherence found between the cognitive control and sensory perception areas of the brain, to possibly imply focused attention on bodily sensations without the need for cognitive reasoning. On the other hand, the increases in conventional, head-surface coherence were hypothesized to possibly indicate increased source strength, as demonstrated by Pascual–Marqui [95]; nonetheless, they also argued for the possibility of other contributing factors besides an increase in source strength.

Therefore, in light of the mounting evidence from EEG studies on the mechanisms of mindfulness, the use of source localization measures to meaningfully clarify varied activity across frequency bands is of paramount importance. Moreover, as it is apparent that neurophysiological findings thus far primarily indicate the implications of mindfulness in attentional processes, the investigation of mindfulness within the fear extinction context, where specific subcortical regions are implied, would require analyses that address such specifications (e.g., the use of LORETA source estimation in [66]).

### 1.4. Implications for Trauma

Echoing the collated neuroimaging evidence on mindfulness and fear extinction [22], the existing state of the EEG literature on mindfulness and fear extinction suggests promising evidence of a relationship between the two constructs, but nonetheless awaits future empirical studies for this relationship to be confirmed. As previously suggested [22], such research efforts would also hold invaluable clinical importance, as it might shed some light on the efficacy of mindfulness-based interventions for clinical disorders such as posttraumatic stress disorder (PTSD) [96,97,98,99,100], specifically where this is characterized by an impaired functioning of the fear extinction network.

Evidence-based treatments that are commonly employed in the treatment of trauma include prolonged exposure, cognitive processing therapy, trauma-focused cognitive behavioral therapy, and eye-movement desensitization therapy, with the relative superiority of any one therapy yet to be definitive [101,102,103,104]. Of particular concern are the high dropout rates that are commonly observed with these trauma-focused approaches, as these patients then continue to suffer from symptoms of PTSD [102,105]. In contrast, King and Favorite [106] noted that most patients (who in this context were veterans) showed high levels of engagement with mindfulness (i.e., MBCT), and had lower dropout rates than what is typically observed with trauma-focused approaches. This could possibly indicate the need for additional strategies, such as emotional regulation and distress tolerance—as cultivated through mindfulness training—to support and promote the engagement of patients in trauma-focused treatment [107,108,109].

As has previously been contended [22], the majority of studies that have found support for the efficacy of mindfulness-based treatments for PTSD are suggestive in nature, such that findings are largely limited to that of self-reported and/or clinician-rated measures of mindfulness, PTSD severity, and/or of secondary measures exploring functional status, general distress, or quality of life [110,111,112,113,114,115,116,117,118,119]. In view of this, the adoption of a multimodal approach (i.e., neurobiological and behavioral measures) is necessary to corroborate existing findings as well as to enable discrepancies between findings to be examined [11,22]. Importantly, the integration of neuropsychological measures in clinical studies could provide valuable information on the mechanisms of the studied mindfulness-based intervention, which could then inform how these interventions can be augmented as a treatment approach for this population.

Thus far, the literature on mindfulness has examined various forms of mindfulness practices and/or interventions, as well as various mindfulness-based meditations under the umbrella term of ‘mindfulness’. This is problematic, as comparisons between varied states, experiences, skills, and practices in mindfulness may yield it difficult to collate findings, and may therefore lead to premature conclusions [11]. For instance, in the neurophysiological literature, Lee et al. [94] suggested mindfulness training to possibly lead to increases in gamma oscillations across multiple brain regions; but additionally, they argued that the specific brain region at which this occurs may depend on the type of meditation delivered. Building on this notion, it is likely that a mindfulness intervention that is targeted toward enhancing the learning and processing of fear extinction may in turn lead to increase in gamma oscillations in brain regions relevant to the fear extinction network, particularly the vmPFC, as described in Mueller et al. [66].

It has also been argued that participation in mindfulness interventions that are not tailored to specific mental health issues (e.g., PTSD) could possibly lead to deteriorations or a worsening of symptoms [11]. Van Dam et al. [11] alluded to the potential risks to participants listed by the MBCT Implementation Resources [120], which include the heightened likelihood of suicide, depression, negative emotions, and intrusive flashbacks amongst trauma patients. It was further made clear that mindfulness practices are not to replace standard psychiatric intervention for trauma, as mindfulness practices still lack clinical studies and evidence that clearly demonstrate their efficacy. Therefore, it is likely that the emerging evidence on the benefits of mindfulness may support its use as an adjunctive treatment instead. Currently, this is noted with the inclusion of mindfulness-based practices as a component in dialetical behavioral therapy for borderline personality disorder, which traditionally has a high rate of trauma history [121]. Beyond this, it should be acknowledged that the research area of mindfulness is relatively still in its infancy; and therefore, its use as a standalone or first-line treatment first necessitates greater research, and is currently limited to the controlled context of clinical studies.

#### 1.4.1. Mindfulness-Based Exposure Therapy

A novel therapy that has recently been introduced is mindfulness-based exposure therapy (MBET), which was developed by a team of clinicians and researchers [108] at the Veterans Affair Ann Arbor, Michigan, US for the treatment of PTSD amongst veterans. The MBET is a 16-week non-trauma focused therapy that incorporates exposure from prolonged exposure therapy, which is one of the standard interventions used with PTSD patients, and is supplemented with mindfulness training from MBCT, self-compassion exercises, and psycho-education on PTSD. In vivo exposures conducted in MBET are conducted with avoided situations/activities that are deemed to be objectively safe, and with no imaginal exposure or processing of trauma histories. On the whole, the intervention consists of four modules: (i) PTSD psycho-education and relaxation strategies, (ii) mindfulness of body and breath exercises and in vivo exposure to feared but objectively safe stimuli (i.e., there is no processing of trauma memories), (iii) mindfulness of emotion and in vivo exposure, and (iv) self-compassion training.

MBET has been trialed in two studies [108,122], and are influential such that they have incorporated pre- to post-neuroimaging measures to corroborate pre- to post-changes in PTSD symptom severity among veterans using the Clinician Administered PTSD Scale (CAPS) [123]. However, instead of fear extinction, investigated changes in brain integrity were particular to social–emotional processing (i.e., the processing of emotional information from faces of other individuals [122]) and the functional connectivity in the default mode network (DMN: the network associated with mind wandering [108]).

As expected, PTSD symptom improvement following MBET was associated with increased activity in the dorsal medial PFC [122] and increases in the DMN (particularly, the posterior cingulate cortex (PCC)) resting state functional connectivity with dorsolateral PFC regions, and that this PCC–dorsolateral PFC connection was correlated with improvement in avoidant and hyperarousal symptoms of PTSD [108] (the findings reported here are those that have been deemed relevant to the aim of this paper. Readers are directed to the original articles of both studies for further results). Together, these findings demonstrate how MBET might be influential for the brain network associated with the emotional regulatory processing of distressing internal experiences during mind wandering.

#### 1.4.2. Neurophysiological Literature on PTSD Using EEG

However, in the neurophysiological literature, no study (at least to our knowledge) has sought to explore the benefits of mindfulness for PTSD using EEG measures. Nonetheless, several reviews [124,125,126,127] have sought to explore the differences in EEG correlates between PTSD and non-PTSD individuals. Specifically, it was found that in comparison to individuals without PTSD, individuals with PTSD demonstrated increased amplitudes in the P50 and P300 family ERPs to aversive stimuli, as well as increased alpha rhythms, and that these increases were correlated with the severity of the posttraumatic stress symptoms (PTSS). Further discussion in the review by Karl et al. [127] suggested the abnormal P300 amplitudes in PTSD to possibly indicate functional changes in the medial frontal–amygdala neural pathways, which as discussed, is implicated in the fear extinction network.

Further support comes from the study by Lee, Yoon, Kim, Jin, and Chung [128], which found decreased connection strength and communication efficiency in gamma and beta activity among individuals with PTSD; these were also significantly correlated with the severity and frequency of PTSD symptoms in general, as well as specific symptoms of re-experiencing and increased arousal. In view of the indication of gamma activity in the extinction of conditioned fear [66], it could be argued that findings by Lee et al. [128] may have reflected non-adaptive fear regulation, which led to increased PTSS, including that of re-experiencing and increased arousal. Building on existing neurophysiological findings of mindfulness (as listed in Table 1), it would therefore be worth exploring the link between mindfulness and fear extinction, and how this link may play a role in altering the relationship between PTSS and its neurophysiological (i.e., EEG) markers.

### 1.5. Future Studies

It is worth reiterating that the use of mindfulness practices in a clinical context still awaits greater studies of methodological rigor. As such—and particular to the area of trauma—future studies are warranted to examine the link between mindfulness and fear extinction for trauma-based symptoms by (i) exploring the pre- to post-changes in brain reactivity to fear-evoking stimuli amongst individuals with PTSD following the delivery of a mindfulness-based intervention, and (ii) to determine if the changes in brain reactivity are associated with changes in posttraumatic stress symptoms from pre- to post-intervention. Drawing from the findings of the extant literature that have been discussed, it is anticipated that participants will demonstrate decreased amplitudes at P300 and LPP ERPs—ERP components, which as discussed, have been identified as relevant to fear regulation when processing emotionally arousing visual stimuli in past studies [50,51,124]. It is also expected that participants will exhibit increased gamma activity—that is, vmPFC, hippocampal [66], and/or amygdala-localized [68]—as well as lowered theta activity [64,65,66].

**Figure 1:**
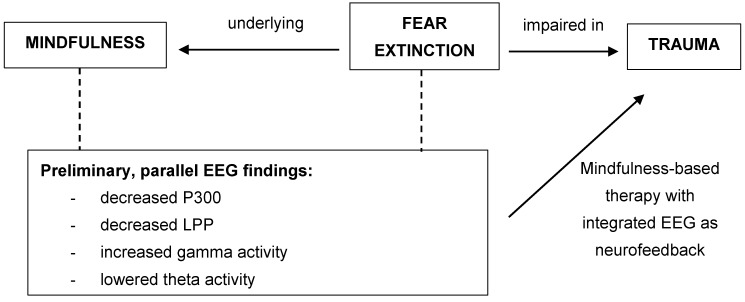
Conceptual connections between mindfulness, fear extinction and trauma, and the suggested implications of electroencephalogram (EEG) as neuromarkers in trauma treatments. LPP—late positive potentials.

Findings from such studies would be especially pertinent to advancing the theoretical knowledge of the link between mindfulness and fear extinction; consequently, they would be of clinical significance on the use of mindfulness-based interventions with clients presenting with PTSS in mental health settings. Moreover, the use of neurophysiological measures in the study could also elucidate its use as a neuromarker for PTSS severity, which may enable earlier intervention and better prognosis and/or prevention of more complex cases of PTSD. Therefore, implications from potential studies would be in line with suggestions by Graham and Milad [129], on using the fear extinction model to enhance the current understanding of treatments for anxiety disorders (e.g., PTSD). They additionally argued that the neural circuits of fear extinction were ideal neuromarkers of symptom severity [129], hence also supporting the integration of neurophysiological measures in future studies to feasibly track neural changes over the course of treatment. See Figure 1.

## 2. Conclusions

As illustrated through this brief review, EEG studies in the integrated research areas of mindfulness and fear extinction are still vastly limited, and we await further studies to build from and to confirm the preliminary findings documented here. This review, consequently, also hopes to have shed some light on the empirical and clinical value of EEG measures in confirming the link between fear extinction and mindfulness. Indeed, the integration of the neuropsychological research areas of mindfulness, fear extinction, and trauma is still in its early conception, but arguably holds invaluable clinical significance that could enhance treatments for fear-based and trauma-related disorders.

## Figures and Tables

**Table 1:** Overview of Neurophysiological Studies on Mindfulness using EEG Discussed in the Present Review. *Note.* State mindfulness = mindfulness induced by meditation; Trait mindfulness = dispositional mindfulness during resting state; ERD = event-related desynchronization; ERS = event-related synchronisation; ERP = event-related potential; OM = open monitoring; FA = focused attention; LPP = late positive potentials; MBCT = mindfulness-based cognitive therapy; MBT = mindfulness-based training; MBSR = mindfulness-based stress reduction. In studies where various meditation styles were examined, the results reported are those relevant to the meditation in bold font.

Study	Sample (Meditators/Control)	Form of Mindfulness/Meditation	EEG Analyses	Findings
Amihai and Kozhevnikov (2014) [72]	10 long-term Theravada meditators (average 8 years of practice)/9 long-term Vajrayana meditators (average 7.4 years of practice)	Theraveda: **Vipassana (OM)**, Kasina (FA); Vajrayana: Deity (OM), Rig-pa (FA)	Spectral power; Coherence	↑ gamma power during Vipassana vs. rest in left hemisphere.
Atchley et al. (2016) [73]	13 non-meditators/15 novice meditators/14 experienced meditators	Mindfulness-based breath counting during tone task	ERP	↑ P3 amplitudes among meditators (novice and experienced) in comparison to non-meditators, in tone-only task (tones as target) ↓ P3 amplitudes among meditators (novice and experienced) in comparison to non-meditators, when engaged in mindfulness-based breath counting during tone task (tones as distractor)
Berkovich-Ohana et al. (2012) [74]	36 mindfulness meditators/12 novice controls	Mindfulness meditation (state mindfulness); Resting state (trait mindfulness)	Spectral power	↓ trait, frontal gamma power (mostly, right lateralized) ↑ trait and state, temporal and parieto-occipital gamma power (mostly, right lateralized)
Berkovich-Ohana et al. (2013) [75]	36 mindfulness meditators/12 novice controls	Mindfulness meditation (state mindfulness); Resting state (trait mindfulness)	Mean Phase Coherence (MPC)	↓ trait, right hemisphere theta MPC ↓ trait, left hemisphere gamma MPC among long-term meditators Negative correlation between trait, left gamma MPC, and meditation expertise
Braboszcz et al. (2017) [76]	20 Vipassana/20 Himalayan Yoga/27 Isha Shoonya/32 Control	**Vipassana**, Himalayan Yoga, Isha Shoonya	Spectral power	↑ parieto-occipital gamma power gamma power positively correlated with meditation experience
Brown et al. (2012) [77]	46 psychology undergraduates (within-subjects design)	Dispositional mindfulness	ERP	↓ LPP amplitude among individuals higher in mindfulness, in response to high arousal, unpleasant pictures
Cahn et al. (2010) [78]	16 long-term meditators (within-subjects design)	Vipassana meditation	Spectral power	↑ frontal theta power ↑ parieto-occipital gamma power occipital gamma power greatest among advanced/ long-term meditators (i.e., 10+ years)
Cahn et al. (2012) [79]	16 long-term meditators (within-subjects design)	Vipassana (mindfulness) meditation	Spectral power; Coherence	↑ gamma power ↑ gamma power in frontal, central, and parietal sites in long-term meditators ↑ frontal gamma coherence (for longer-term meditators) ↑ theta coherence
Delgado-Pastor et al. (2013) [80]	10 experienced meditators (within-subjects design)	Vipassana (mindfulness) meditation	ERP	↑ P3b amplitudes to a target tone
Eddy et al. (2015) [81]	24 participants (within-subjects design)	Induced mindfulness (through focused breathing)	ERP	Induced mindfulness (i.e., decentering) correlated with ↓ P300 amplitudes for negative vs. neutral images
Egan et al. (2017) [82]	118 adult sample (within-subjects design)	Brief mindfulness instructions	ERP	↑ LPP amplitude in brief mindfulness instructions
Hauswald et al. (2015) [83]	11 meditators (within-subjects design)	Zen	Spectral power	↑ gamma power ↓ frontal theta power
Lakey et al. (2011) [84]	9 naïve meditators/9 control	Short, 6 minutes mindfulness induction	ERP	↑ P300 amplitude in central and temporal electrodes
Lehmann et al. (2012) [85]	13 Tibetan Buddhists/15 QiGong practitioners/14 Sahaja Yoga practitioners/14 Ananda Marga Yoga practitioners/15 Zen practitioners	Various (including **Zen**)	Spectral power; Lagged intracortical coherence; Head-surface conventional coherence	↑ gamma power during meditation vs. resting as averaged across groups ↑ theta head-surface conventional coherence in meditation vs. initial resting in Zen ↓ lagged intracortical coherence during meditation vs. resting (for all meditation tradition) in all frequency bands
Lutz et al. (2004) [86]	8 long-term Buddhist practitioners/10 healthy student volunteers	Loving kindness and compassion-focused meditation	Spectral power; Coherence	↑ gamma power in bilateral pareito-temporal and midfrontal electrodes ↑ ratio of gamma power to low frequency power (4–13 Hz: Theta and Alpha) at medial frontoparietal electrodes at initial baseline; at frontolateral and posterior electrodes during meditation; and at anterior electrodes at post-meditative baseline ↑ size of gamma coherence over lateral frontoparietal electrodes Positive correlation between hours of meditation and gamma power at initial baseline
Miltz et al. (2014) [87]	23 naïve meditators (within-subjects design)	Breath counting (indicative of a meditative state)	Spectral power; Lagged intracortical coherence; Head-surface conventional coherence	↓ theta intracortical lagged coherence between left middle frontal gyrus and right inferior parietal lobule ↓ theta intracortical lagged coherence between left middle frontal gyrus and right cunues ↑ gamma head-surface conventional coherence within the left anterior region
Slagter et al. (2007) [88]	17 participants/23 control (mixed design)	3-month meditation (Vipassana) retreat	ERP	↓ P3b amplitude to distractor stimuli
Sobolewski et al. (2011) [89]	13 meditators/13 control	Mindfulness meditation	ERP	↓ LPP amplitude in frontal scalp regions to negatively valence visual stimuli
van Leeuwen et al. (2012) [90]	8 Buddhist monks and nuns/8 control 8 experienced FA meditators/6 controls (mixed design)	Zen (FA and OM meditation practices) 4-day OM meditation retreat	ERP ERP	↑ P300 amplitude in processing of small-sized targets (which is embedded within larger targets) in meditators only In the processing of larger-sized targets, ↑ P300 amplitude in both meditators and control Decreased attention processing towards detailed (small) targets from pre-retreat to post-retreat.
Wong et al. (2018) [91]	36 nurses (longitudinal design)	8-week MBT (based on MBSR)	Spectral power; ERP	↑ P300 amplitude
